# Flavor Characteristics of Ganpu Tea Formed During the Sun-Drying Processing and Its Antidepressant-Like Effects

**DOI:** 10.3389/fnut.2021.647537

**Published:** 2021-03-24

**Authors:** Sui Xiao, Jingyuan Huang, Yahui Huang, Huiqing Lai, Yi Zheng, Dahua Liang, Hang Xiao, Xu Zhang

**Affiliations:** ^1^Guangdong Key Laboratory for Innovative Development and Utilization of Forest Plant Germplasm, College of Forestry and Landscape Architecture, South China Agricultural University, Guangzhou, China; ^2^Department of Food Science, University of Massachusetts, Amherst, MA, United States; ^3^Department of Tea Science, College of Horticulture, South China Agricultural University, Guangzhou, China; ^4^Yunding Ganpu Tea Industry Co., LTD, Guangzhou, China

**Keywords:** Ganpu tea, flavor, sun-drying processing, antidepressant-like properties, health functions

## Abstract

Ganpu tea is a novel type of tea beverage with unique and pleasant flavor that encases Pu-erh tea leaves within an intact mandarin peel. However, to date, no holistic and detail studies on its chemical composition and biological activities have been reported yet. In the present study, by applying UPLC-Q-TOF and UPLC-MS technology, we systematically identified and analyzed 104 water-soluble compounds of Ganpu tea and their variation trend during the sun-drying processing. The results showed that the generation of pigments and gallic acid coincided with a dramatic decrease in catechin content, and a significant increase in alkaloid and flavonoid contents. The conversion of these compounds can contribute to the improvement of sensory attributes of Ganpu tea and maybe indispensable to its unique flavor. Moreover, the mice given orally with high dose of Ganpu tea (0.4 g/kg) showed a significantly reduced immobility duration as compared to that of the negative control group (*p* < 0.01) both in the forced swimming test and tail suspension test. Together, these results indicate that the sun-drying processing was indispensable to the formation of the unique flavor for Ganpu tea. Multiple types of compounds of Ganpu tea may collectively provide the synergistic attributes to its antidepressant-like properties.

## Introduction

Ganpu tea is a novel type of tea beverage and has gained considerable popularity in China since 2015 due to its potential health-promoting effects and unique flavor. The production value of Ganpu tea in 2016 was ~1.6 billion RMB, almost a 3-fold increase as compared to that in 2015 (1). Due to the rapidly increasing consumption of Ganpu tea, over 2,000 new Ganpu tea enterprises have been registered in Xinhui city, Guangdong in 2017. The consumption of a combination of tea leaves and citrus peel as brewed tea can be dated back to the eighth century in Tang dynasty. It is also well-known that mandarin peel can be used as an ingredient in traditional Chinese medicine as well as functional foods (2, 3). Nowadays, the contemporary Ganpu tea product is made with both Pu-erh tea and mandarin peel. The processing of Ganpu tea involves (a) the removal of pulp from the fruit peel while keeping the peel intact; (b) cleaning the peel and filling it with Pu-erh tea; and (c) finally sun-drying and packaging.

Pu-erh tea can be divided into Pu-erh raw tea and Pu-erh ripen tea, depending on the processing techniques and quality characteristics. For the production of Pu-erh ripen tea, the sun-dried green tea is generally used as a substrate for microbial fermentation under the high temperature and humidity conditions (4). Due to pretense of a large number of active microorganisms in this processing, such as *Aspergillus niger, Penicllium, Rhizopus*, and *Aspergillus gloucus* (5), production of Pu-erh ripen tea will face considerable risk to food safety if it is used as the ingredient for Ganpu tea. Conversely, Pu-erh raw tea is made directly from the sun-dried green tea and does not include a microbial fermentation step, and therefore, the chemical constituents and quality are more similar to those of the sun-dried green tea than to those of Pu-erh ripen tea (6). In the present study, Pu-erh raw tea was used as the ingredient for production of Ganpu tea, due to not only its relatively high food safety, but also its flavor profile being relatively well-matched with those of mandarin peel. The mandarin peel is a popular product with strong geographical indication, as the most authentic production areas are confined to the specific regions in Xinhui, Guangdong, China. Citrus peels are rich in various functional components and enzymes, which catalyze the conversion of major chemical compounds during the sun-drying processing, and provide a fruity flavor to Ganpu tea.

To date, numerous methods including hot-air drying, freeze drying, vacuum drying, and infrared drying, have been applied to dry agricultural products (7–9). Hot-air drying is the method most commonly used to dry Ganpu tea. This method has the advantages of higher efficiency, lower cost, and easier control over the external odor infiltration. However, the quality of Ganpu tea dried with this technique is questionable because the hot-air drying can impact the thermosensitive substances in the mandarin peel, causing them to crack over time. In contrast, the sun-drying method has a more suitable temperature and humidity for aging, with ample time for development and improvement of the potential flavor compounds in Ganpu tea.

In the present study, we processed Ganpu tea from both Pu-erh raw tea and mandarin peel using the sun-drying technique. Pu-erh raw tea and mandarin peel have been studied extensively in the functional drink and health product fields, respectively (4, 10–13). However, as a recently popularized commodity, to date, no holistic and detailed studies on its chemical characteristics and biological activities of Ganpu tea have been reported yet. We previously analyzed the aromatic substances of Ganpu tea and discovered a number of volatile compounds that were newly generated during the sun-drying processing (14). In this study, we systematically identified and analyzed the water-soluble compositions of Pu-erh raw tea, mandarin peel, and Ganpu tea by applying the UPLC-Q-TOF-MS and UPLC-MS technology, and investigated the conversions of potential flavor compounds of Ganpu tea during the sun-drying processing. We also conducted formal sensory evaluation to assess the final influence of these compositional changes on flavor quality criteria. Additionally, we also evaluated the preventive effects of Ganpu tea extract on depression pathology both in the FST and TST mice models. Collectively, our experiments aimed to reveal the possible chemical basis for the flavor formation of Ganpu tea and to demonstrate whether these compounds can exert antidepressant effects.

## Materials and Methods

### Plant Materials

The tea plant materials were collected from Yunding Ganpu tea industry co., LTD (Guangzhou, China). Information regarding the tea plant samples used in this study are described in Table 1. S1, S2, and S3 were prepared to compare the chemical compositions among Pu-erh raw tea, mandarin peel, and Ganpu tea. S4, S5, S6, and S7 were prepared for research on the variation trend of potential flavor compounds in Ganpu tea during the sun-drying processing. All samples were tested in time on the harvesting year.

**Table 1:** Sample information sheet.

**Sample No.**	**Sample name**	**Description**	**Collection time**
S1	Pu-erh raw tea	Harvested from Yunnan	2016.12
S2	Mandarin peel	Harvested from Xinhui, Guangdong	2016.12
S3	Ganpu tea	Made from S1 and S2	2016.12
S4	Ganpu tea 0 day	0 day of the full sun-drying processing	2018.09
S5	Ganpu tea 3 day	3 day of the full sun-drying processing	2018.09
S6	Ganpu tea 9 day	9 day of the full sun-drying processing	2018.09
S7	Ganpu tea 15 day	15 day of the full sun-drying processing	2018.09

### Chemicals and Drugs

HPLC-grade acetonitrile, methanol, and formic acid were purchased from Fisher Scientific (Pittsburg, PA, USA). Water was purified using a Milli-Q water purification system (Millipore, Bedford, MA, USA). Clomipramine hydrochloride tablets were purchased from Beijing Novartis Pharmaceutical Co., Ltd (Beijing, China). All the other chemicals and reagents used were of the highest grade available.

### Preparation of Samples

Tea plant samples were dried to constant weight using a vacuum freeze-drying machine, and the remaining weight was milled into a powder with ~60 meshes. Approximately 2.0 g of the prepared powder sample was weighed and extracted with 100 mL of distilled water at 100°C for 45 min with shaking, and the extracts were further filtered through filter paper. The obtained supernatants were filtered through a 0.22 um membrane for subsequent UPLC-Q-TOF and UPLC-MS analysis, and then dried into powder by vacuum freeze-drying machine. The dried extract powder was subsequently used in antidepressant-like testing in mice described below (15).

### UPLC-Q-TOF Analysis

An Agilent UPLC1290-6540-UHD Q-TOF system and an ACQUITY UPLC BEH C18 column (2.1 mm × 100 mm id, 1.7 μm, Waters) were used for the qualitative analysis. The mobile phase was composed of A (0.2% formic acid, v/v) and B (acetonitrile) with a gradient elution as follows: 0–15 min, 95%−95% A; 15–35 min, 95%−85% A; 35–50 min, 85%−50% A; 50–60 min, 50%−20% A; and 60–65 min, 20%−95% A. The column and auto-sampler temperatures were maintained at 40 and 20°C, respectively. The mobile phase was directly delivered into the electrospray ionization source at a flow rate of 0.3 mL/min. The injection volume was 5 μL. Mass spectroscopy (MS) analysis was performed in both positive and negative ion mode with the full scan mode from m/z 0 to 1,200. The capillary and cone voltages were 3.5 kV and 30 V, respectively. The collision energy was 40 eV. Nitrogen was used as desolvation gas at flow rate of 500 L/h. The temperatures of ion source and desolvation gas were set at 100 and 300°C, respectively.

### UPLC-MS Analysis

An Waters ACQUITY™ QDA system coupled with PDA detector (Waters, Milford, MA, USA) were used for further research on the flavor characteristics of Ganpu tea. The chromatographic parameters, including column, mobile phase, gradient elution condition, flow rate, and injection volume, were the same as UPLC-Q-TOF-MS system described in 2.4. The UV chromatogram was monitored at 278 nm. MS analysis was performed both in positive and negative ion mode and using the full scan mode from m/z 50 to 1,200. The capillary and cone voltages were 3.5 kV and 25 V, respectively.

### Analysis of Biochemical Compositions

The contents of moisture, water extract, total free amino acids and tea polyphenols were determined according to GB 5009.3-2016, GB/T 8305-2013, GB/T8314-2013, and GB/T 8313-2008, respectively. The content of tea pigment was determined using the method described in our previous work with proper modifications (15).

### Descriptive Sensory Analysis (DSA)

The tea infusion was evaluated by a well-trained panel of 10 members (five males and five females, age: 20–45). Tea samples were assessed using the flavor profile method of ISO 6564 (ISO, 1985). The DSA was performed according to the procedures described previously by Kraujalyt et al. (16) and GB/T 14487-2017, with a slight modification. Briefly, 3.00 g of Ganpu tea was infused with 150 mL of freshly boiled water for 5 min in a specialized DSA tea pot, then 50 mL of tea infusion was poured into a 150 mL-cup covered with a porcelain cover, which was provided to panelists in an odor-free laboratory at 25°C. Three-digit numbers were used to code samples, and they were randomly offered to panelists after brewing for aroma and flavor assessment. Panelists agreed that the samples could be described using six attributes for aroma: stale, woody, floral, fruity, sweet, and off-flavor, as well as six attributes for flavor: bitter, silky, sweet, umami, mellow, and astringent. The intensities of the aroma and flavor attributes were scored using a scale from 0 to 10, where 0 = none or negligible perceptible intensity, and 10 = extremely high intensity. Each sample was evaluated three times by each panelist on different days. Data were expressed as a mean.

### Animals

Both male and female Chinese Kun Ming (KM) mice (20 ± 2 g) were obtained from Laboratory Anima Center of Guangzhou University of Chinese Medicine (Guangzhou, Guangdong Province, China) and used in this study. The mice were housed individually in cages for 3 days to adapt to the environment under the following controlled conditions: 25 ± 2°C under a 12 h light/dark cycle and 60 ± 5% humidity with free access to water and food pellets. Each mouse was used only once. Behavioral testing was performed between 7:00 and 12:00 AM. All the procedures were conducted according to the National Institutes of Health Guide for the Care and Use of Laboratory Animals, and the animal handling and experimental procedures were approved by the Local Animal Use Committee (SCXK 2013-0020).

### Measurement of Depressive-Like Behaviors

Behavior tests were performed in five groups with 12 mice in each group. The mice were given orally twice a day with the extract of Ganpu tea at 0.1 g /kg, 0.2 g /kg, and 0.4 g /kg for 15 consecutive days, respectively. Mice in positive control group were given with clomipramine hydrochloride tablets (0.02 g /kg) as the positive reference also for 15 consecutive days. The mice in negative control group were given only with the same volume of physiological saline solution for 15 consecutive days. The mice in both positive and negative control groups were tested in parallel with those receiving various doses of extract of Ganpu tea. Animal behaviors were assessed at 45 min after administration of the final dose.

#### Forced Swimming Test (FST)

The FST is one of the most common animal models used for examination of the depressive-like behaviors. The test was conducted as previously described (17) with slight modifications. Mice were individually inserted in an open cylinder container (10 cm in diameter and 25 cm in height) containing 19 cm of water at 24 ± 2°C. Each mouse was judged to be immobile when it ceased struggling and remained floating motionless in the water, making only small movements necessary to keep its head above water. The duration of immobility was recorded during the last 5 min of the 7 min testing period.

#### Tail Suspension Test (TST)

The TST is another model commonly used for examination of depressive-like behaviors (18). The tails of mice were taped in a balance bracket at a distance of 2 cm from the tail tip, with tail straight and head being hung down 15 cm above the desktop after 45 min from the last administration. The mice were separated by cardboard just in the case when they were interfered with each other. Each mouse was treated for 7 min; the first 2 min was for adaptation. The duration of immobility (no other body movements except respiration) in the last 5 min was recorded.

### Statistical Analysis

UPLC-Q-TOF-MS and UPLC-MS system were re-calibrated after analysis of every 5 samples to ensure and maintain data accuracy and stability. All the results were recorded and expressed as mean ± standard deviation (SD) of three replicates. The Heatmap analysis was carried out using the website https://software.broadinstitute.org/morpheus, and based on the relative abundances by untargeted metabolomics. The statistical analyses were performed using one-way analysis of variance (ANOVA), followed by least significant difference (LSD) test or Duncan's test. *p* < 0.05 was considered statistically significant.

## Results and Discussion

### Comparisons of Chemical Compositions Among Pu-erh Raw Tea, Mandarin Peel and Ganpu Tea

**Figure 1:**
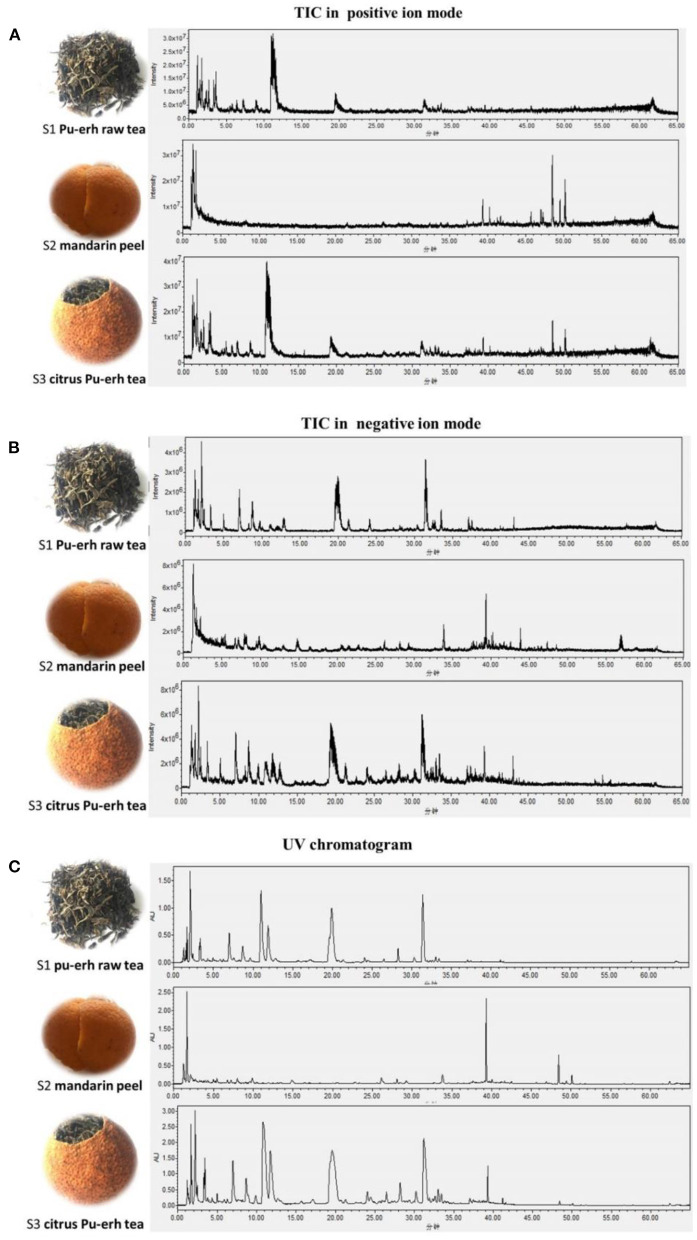
Comparisons of the chemical compositions among Pu-erh raw tea, mandarin peel, and Ganpu tea by TIC in positive ion mode **(A)**, TIC in negative ion mode **(B)**, and UV chromatogram **(C)**, respectively. In our previous study, we investigated the source and conversion of aromatic substances of Ganpu tea. The fruity sweet aromas compounds of Ganpu tea were consisted of the main volatile components of Pu-erh raw tea and mandarin peel, in addition to the newly generated compounds during the sun-drying processing (14). Based on the data obtained from the present study, we could draw a similar conclusion that the water-soluble substances of Ganpu tea were not just a simple combination of the compounds of Pu-erh raw tea and mandarin peel. Rather, the water-soluble substances of Ganpu tea were mainly attributed to the development and transformation of the precursor ingredients that it contains and to several newly generated substances during the sun-drying processing. The final formation of the flavor of Ganpu tea may be closely related to the production and conversion of these water-soluble substances. Further researches are necessary to investigate the contribution of these compounds to the flavor characteristic of Ganpu tea.

After being extracted with boiling water, the extract was filtered and injected into the UPLC-Q-TOF system and the UPLC-MS system, respectively. Good separation was achieved after screening a series of mobile phases, UPLC columns, and gradient profiles. Meanwhile, MS conditions were optimized after testing a set of instrument parameters, as described in UPLC-Q-TOF Analysis and UPLC-MS Analysis. The total ion chromatograms (both positive and negative ion modes) and the representative UPLC chromatograms (278 nm) of Pu-erh raw tea (S1), mandarin peel (S2), and Ganpu tea (S3) were obtained by the UPLC-MS system (Figure 1). Peak identification of compounds in Table 2 was performed based on the precise molecular weight and fragment ions by UPLC-Q-TOF analysis, and a comparison of them with data in the previously published literature (19–23).

**Table 2:** List of the identified components in tested samples.

**Peak No.**	**Putative identification**	**Rt (min)**	**[M+H]^+^/[M-H]^−^**	**Measured value**	**Fragment ions**	**Formula**	**Detection**						
							**S1**	**S2**	**S3**	**S4**	**S5**	**S6**	**S7**
1	Theanine	1.7	[M+H]+	175.1082	158, 129	C_7_H_14_N_2_O_3_	✓	_	✓	✓	✓	✓	✓
2	Leucine	2.1	[M+H]+	132.1012	132, 86	C_6_H_13_NO_2_	✓	_	✓	✓	✓	✓	✓
3	Phenylalanine	3.28	[M+H]+	166.0863	120	C_9_H_11_NO_2_	✓	_	✓	✓	✓	✓	✓
4	Tryptophan	6.3	[M+H]+	205.0974	143, 118	C_11_H_12_N_2_O_2_	✓	_	✓	✓	✓	✓	✓
5	Tyrosine	3.05	[M+H]+	182.0808	165, 136	C_9_H_11_NO_3_	✓	_	✓	✓	✓	✓	✓
6	Pyroglutamic acid	2.6	[M+H]+	130.0501	_	C_5_H_7_NO_3_	✓	_	✓	✓	✓	✓	✓
7	* Isoleucine	2.3	[M+H]+	132.1013	86	C_6_H_13_NO_2_	_	_	✓	_	_	✓	✓
8	* Threonine	3.2	[M+H]+	120.0650	_	C_4_H_9_NO_3_	_	_	✓	_	_	✓	✓
9	* Arginine	1.45	[M+H]+	175.1120	158, 129	C_6_H_14_N_4_O_2_	_	_	✓	_	_	✓	✓
10	* Glutamine	8.25	[M+H]+	147.0758	119, 91	C_5_H_10_N_2_O_3_	_	_	✓	_	_	✓	✓
11	Gallocatechin (GC)	3.35	[M+H]+	307.0813	_	C_15_H_14_O_7_	✓	_	✓	✓	✓	✓	✓
12	(-)-Epigallocatechin (EGC)	7.00	[M+H]+	307.0814	223, 163	C_15_H_14_O_7_	✓	_	✓	✓	✓	✓	✓
13	Catechin (C)	8.7	[M+H]+	291.0869	_	C_15_H_14_O_6_	✓	_	✓	✓	✓	✓	✓
14	Epicatechin (EC)	19.4	[M+H]+	291.0863	165, 139	C_15_H_14_O_6_	✓	_	✓	✓	✓	✓	✓
15	Epigallocatechin gallate (EGCG)	19.8	[M+H]+	459.0922	289, 139	C_22_H_18_O_11_	✓	_	✓	✓	✓	✓	✓
16	Gallocatechin gallate (GCG)	24.0	[M+H]+	459.0924	289, 139	C_22_H_18_O_11_	✓	_	✓	✓	✓	✓	✓
17	Epicatechin gallate (ECG)	31.3	[M+H]+	443.0972	273, 139	C_22_H_18_O_10_	✓	_	✓	✓	✓	✓	✓
18	Catechin gallate (CG)	32.6	[M+H]+	443.0973	_	C_22_H_18_O_10_	✓	_	✓	✓	✓	✓	✓
19	(-)-EGCG-3″-O-ME	22.6	[M-H]-	471.0920	_	C_23_H_20_O_11_	✓	_	✓	✓	✓	✓	✓
20	Epiafzelechin	3.00	[M+H]+	275.0919	_	C_15_H_14_O_5_	✓	_	✓	✓	✓	✓	✓
21	Epiafzelechin-3-O-gallate	37.2	[M-H]-	424.0795	273	C_22_H_17_O_9_	✓	_	✓	✓	✓	✓	✓
22	Gallocatechin-(4a-8)-catechin-3-O-gallate	20.7	[M-H]-	745.1399	457, 423	C_37_H_30_O_17_	✓	_	✓	✓	✓	✓	✓
23	Digallocatechin-catechin	25.9	[M-H]-	897.1869	423	C_45_H_38_O_20_	✓	_	✓	✓	✓	✓	✓
24	*Catechin-4a-epicatechin-3-O-gallate	24.5	[M+H]+	731.1608	427, 289	C_37_H_30_O_16_	_	_	✓	_	✓	✓	✓
25	Trigalloylglucose	28.25	[M-H]-	635.0880	423, 169	C_27_H_24_O_18_	✓	_	✓	✓	✓	✓	✓
26	*Catechin-4a-epicatechin 3'-o-gallate	26.5	[M+H]+	731.1608	427, 289	C_37_H_30_O_16_	_	_	✓	_	✓	✓	✓
27	Theasinesnsins A	32.00	[M+H]+	611.1395	465,303	C_30_H_26_O_14_	✓	_	✓	✓	✓	✓	✓
28	Theasinesnsins B	33.1	[M+H]+	611.1396	465, 303	C_30_H_26_O_14_	✓	_	✓	✓	✓	✓	✓
29	Caffeine	11.0	[M+H]+	195.0877	157	C_8_H_10_N_4_O_2_	✓	_	✓	✓	✓	✓	✓
30	Theobromine	3.45	[M+H]+	181.0721	138, 108	C_7_H_8_N_4_O_2_	✓	_	✓	✓	✓	✓	✓
31	Theophylline	5.3	[M+H]+	181.0718	_	C_7_H_8_N_4_O_2_	✓	_	✓	✓	✓	✓	✓
32	Theaflavin	30.0	[M-H]-	563.1190	_	C_29_H_24_O_12_	✓	_	✓	✓	✓	✓	✓
33	^*^Procyanidin B1	15.17	[M+H]+	579.1497	409, 289	C_30_H_26_O_12_	✓	_	✓	✓	✓	✓	✓
34	^*^Procyanidin B2	15.26	[M+H]+	579.1497	409, 289	C_30_H_26_O_12_	✓	_	✓	✓	✓	✓	✓
35	^*^Procyanidin B3	15.7	[M+H]+	579.1495	409, 127	C_30_H_26_O_12_	✓	_	✓	✓	✓	✓	✓
36	^*^Procyanidin B4	15.76	[M+H]+	579.1497	409, 289	C_30_H_26_O_12_	✓	_	✓	✓	✓	✓	✓
37	Procyanidin B1 3-O-gallate	24.5	[M+H]+	731.1608	427, 289	C_37_H_30_O_16_	✓	_	✓	✓	✓	✓	✓
38	Procyanidin B2 3-O-gallate	26.5	[M+H]+	731.1608	427, 289	C_37_H_30_O_16_	✓	_	✓	✓	✓	✓	✓
39	Rutin	41.0	[M+H]+	611.1606	465, 303	C_27_H_30_O_16_	✓	_	✓	✓	✓	✓	✓
40	Kaempferol	37.5	[M+H]+	287.0552	_	C_15_H_10_O_6_	✓	_	✓	✓	✓	✓	✓
41	^*^Kaempferol-3-O-glucoside	35.91	[M-H]-	447.0929	284, 255	C_21_H_20_O_11_	✓	_	✓	✓	✓	✓	✓
42	^*^Kaempferol-3-O-galactoside	37.56	[M-H]-	447.0925	284, 255	C_21_H_20_O_11_	✓	_	✓	✓	✓	✓	✓
43	^*^Kaempferol-3- O-rutinoside	37.1	[M-H]-	593.1509	_	C_27_H_30_O_15_	✓	_	✓	✓	✓	✓	✓
44	^*^Kaempferol-3- O-glucosylrutinoside	35.6	[M-H]-	755.2031	_	C_33_H_40_O_20_	✓	_	✓	✓	✓	✓	✓
45	^*^Myricetin-3-O-glucoside	27.2	[M-H]-	479.0824	_	C_21_H_20_O_13_	✓	_	✓	✓	✓	✓	✓
46	^*^* Myricetin−3- locust glycoside	28.1	[M+H]+	625.1036	_	C_26_H_24_O_18_	_	_	✓	_	✓	✓	✓
47	^**^Myricetin-3-O-galactoside	28.05	[M-H]-	479.0827	_	C_21_H_20_O_13_	✓	_	✓	✓	✓	✓	✓
48	Quercetin	30.3	[M+H]+	303.0500	287, 257	C_15_H_10_O_7_	✓	_	✓	✓	✓	✓	✓
49	^*^* Quercetin−3-o- rhamnoside galactoside	39.35	[M+H]+	611.1250	303	C_26_H_26_O_17_	_	_	✓	_	✓	✓	✓
50	^*^Quercetin**-3-O-glucoside**	32.4	[M-H]-	463.0877	300, 271	C_21_H_20_O_12_	✓	_	✓	✓	✓	✓	✓
51	^*^Quercetin**-3-O-galactoside**	33.5	[M-H]-	463.0878	300, 271	C_21_H_20_O_12_	✓	_	✓	✓	✓	✓	✓
52	^*^Quercetin−3- O-glucosylrutinoside	31.85	[M+H]+	773.2136	303, 465	C_33_H_40_O_21_	✓	_	✓	✓	✓	✓	✓
53	* Vitexin	49.5	[M+H]+	433.1129	395	C_21_H_20_O_10_	_	_	✓	_	✓	✓	✓
54	Gallic acid	2.25	[M-H]-	169.0142	125	C_7_H_6_O_5_	✓	_	✓	✓	✓	✓	✓
55	Quinic acid	1.3	[M-H]-	191.0560	111, 87	C_7_H_12_O_6_	✓	_	✓	✓	✓	✓	✓
56	Caffeic acid	2.21	[M+H]+	181.0500	157	C_9_H_8_O_4_	✓	_	✓	✓	✓	✓	✓
57	Malic acid	1.4	[M-H]-	133.0137	75	C_4_H_6_O_5_	✓	_	✓	✓	✓	✓	✓
58	Ascorbic acid	1.9	[M-H]-	175.0240	115, 85	C_6_H_8_O_6_	✓	_	✓	✓	✓	✓	✓
59	Caffeoylmalic acid	3.3	[M-H]-	295.0442	191, 113	C_13_H_12_O_8_	✓	_	✓	✓	✓	✓	✓
60	^*^3-O-galloylquinic acid	2.15	[M-H]-	343.0662	191, 169	C_14_H_16_O_10_	✓	_	✓	✓	✓	✓	✓
61	^*^5-O-galloylquinic acid	2.48	[M-H]-	343.0664	191, 169	C_14_H_16_O_10_	✓	_	✓	✓	✓	✓	✓
62	Chlorogenic acid	5.0	[M-H]-	353.0870	191	C_16_H_18_O_9_	✓	_	✓	✓	✓	✓	✓
63	^*^1-Caffeoylquinic acid	10.00	[M-H]-	353.0871	191	C_16_H_18_O_9_	✓	_	✓	✓	✓	✓	✓
64	^*^3-Caffeoylquinic acid	10.09	[M-H]-	353.0869	291, 191	C_16_H_18_O_9_	✓	_	✓	✓	✓	✓	✓
65	^*^1-p-Coumaroylquinic acid	16.01	[M-H]-	337.0915	305, 163	C_16_H_18_O_8_	✓	_	✓	✓	✓	✓	✓
66	^*^3-p-Coumaroylquinic acid	16.3	[M-H]-	337.0917	305, 163	C_16_H_18_O_8_	✓	_	✓	✓	✓	✓	✓
67	^*^4-p-Coumaroylquinic acid	16.87	[M-H]-	337.0918	305, 163	C_16_H_18_O_8_	✓	_	✓	✓	✓	✓	✓
68	^*^5-p-Coumaroylquinic acid	16.92	[M-H]-	337.0918	305, 163	C_16_H_18_O_8_	✓	_	✓	✓	✓	✓	✓
69	Galloylglucose	45.5	[M-H]-	331.0662	169	C_13_H_16_O_10_	✓	_	✓	✓	✓	✓	✓
70	1,2,6-Trigalloylglucose	28.2	[M-H]-	635.0882	465, 313	C_27_H_24_O_18_	✓	_	✓	✓	✓	✓	✓
71	3,6-Digalloylglucose	9.9	[M-H]-	483.0773	169	C_20_H_20_O_14_	✓	_	✓	✓	✓	✓	✓
72	Strictinin	11.8	[M-H]-	633.0725	301, 275	C_27_H_22_O_18_	✓	_	✓	✓	✓	✓	✓
73	Synephrine	1.66	[M+H]+	168.1012	150, 135	C_9_H_13_NO_2_	_	✓	✓	✓	✓	✓	✓
74	Lucenin-2	3.691	[M+H]+	611.1609	575, 473	C_27_H_30_O_16_	_	✓	✓	✓	✓	✓	✓
75	Naringenin	23	[M+H]+	273.0757	274, 202	C_15_H_12_O_5_	_	✓	✓	✓	✓	✓	✓
76	Vicenin-2	37	[M+H]+	595.1658	457, 409	C_27_H_30_O_15_	_	✓	✓	✓	✓	✓	✓
77	Diosmetin-6, 8-di-C-glucoside	28.1	[M+H]+	625.1762	607, 589	C_28_H_32_O_16_	_	✓	✓	✓	✓	✓	✓
78	Naringin	37.75	[M+H]+	581.1864	_	C_27_H_32_O_14_	_	✓	✓	✓	✓	✓	✓
79	Chysoeriol-6, 8-di-C-glucoside	29.2	[M+H]+	625.1766	409, 355	C_28_H_32_O_16_	_	✓	✓	✓	✓	✓	✓
80	Diosmetin-6-C-glucoside	35.8	[M+H]+	463.1236	367, 343	C_22_H_22_O_11_	_	✓	✓	✓	✓	✓	✓
81	Narirutin	37.7	[M-H]-	579.1711	271	C_27_H_32_O_14_	_	✓	✓	✓	✓	✓	✓
82	Rhoifolin	12.0	[M+H]+	579.1709	433, 271	C_27_H_30_O_14_	_	✓	✓	✓	✓	✓	✓
83	Diosmin	39.2	[M+H]+	609.1816	463, 301	C_28_H_32_O_15_	_	✓	✓	✓	✓	✓	✓
84	Hesperidin	39.35	[M-H]-	609.1818	343, 301	C_28_H_34_O_15_	_	✓	✓	✓	✓	✓	✓
85	Poncirin	26.1	[M+H]+	595.2023	463, 379	C_28_H_34_O_14_	_	✓	✓	✓	✓	✓	✓
86	Citrusin III	43.8	[M+H]+	728.3982	700, 587	C_36_H_53_N_7_O_9_	_	✓	✓	✓	✓	✓	✓
87	Melitidin	44.5	[M+H]+	725.2293	419, 404	C_33_H_40_O_18_	_	✓	✓	✓	✓	✓	✓
88	Monohydroxy-tetramethoxyflavone	48.95	[M+H]+	359.1125	329, 301	C_19_H_18_O_7_	_	✓	✓	✓	✓	✓	✓
89	Pentamethoxyflavanone	50.15	[M+H]+	375.1441	211, 196	C_20_H_22_O_7_	_	✓	✓	✓	✓	✓	✓
90	Monohydroxy-pentamethoxyflavone	51.2	[M+H]+	389.1231	374, 359	C_20_H_20_O_8_	_	✓	✓	✓	✓	✓	✓
91	Isosinensetin	45.6	[M+H]+	373.1284	343, 153	C_20_H_20_O_7_	_	✓	✓	✓	✓	✓	✓
92	Sinensetin	47	[M+H]+	373.1283	343, 153	C_20_H_20_O_7_	_	✓	✓	✓	✓	✓	✓
93	Tetramethyl-O-isoscutellarein	47.2	[M+H]+	343.1176	328, 313	C_19_H_18_O_6_	_	✓	✓	✓	✓	✓	✓
94	Limonin	43	[M+H]+	471.2015	425, 397	C_26_H_30_O_8_	_	✓	✓	✓	✓	✓	✓
95	Nobiletin	48.5	[M+H]+	403.1386	373, 211	C_21_H_22_O_8_	_	✓	✓	✓	✓	✓	✓
96	Tetramethyl-O-scutellarein	48.6	[M+H]+	343.1175	327, 313	C_19_H_18_O_6_	_	✓	✓	✓	✓	✓	✓
97	3, 5, 6, 7, 8, 3′, 4′-Heptamethoxyflavone	49.5	[M+H]+	433.1496	403, 388	C_22_H_24_O_9_	_	✓	✓	✓	✓	✓	✓
98	Tangeretin	50.2	[M+H]+	373.1285	358, 343	C_20_H_20_O_7_	_	✓	✓	✓	✓	✓	✓
99	5-Hydroxy-6, 7, 8, 3′, 4′-pentamethoxyflavone	46.1	[M+H]+	389.1231	_	C_20_H_20_O_8_	_	✓	✓	✓	✓	✓	✓
100	5-Hydroxy-4′- methoxy flavone-7-o-glycoside	37.5	[M+H]+	449.1440	287	C_22_H_24_O_10_	_	✓	✓	✓	✓	✓	✓
101	*4H-1-Benzopyran-4-one,2,3-dihydro-5,6,7,8-tetramethoxy-2-[4-(phenylmethoxy)phenyl]-	44.45	[M+H]+	725.1870	_	C_39_H_32_O_14_	_	_	✓	_	_	✓	✓
102	4H-1-Benzopyran-4-one,6,8-di-b-Dglucopyranosyl-5,7-di-hydroxy-2-(4-hydroxy-3-me-thoxyphenyl)-	28.1	[M+H]+	625.1765	_	C_28_H_32_O_16_	_	✓	✓	✓	✓	✓	✓
103	4H-1-Benzopyran-4-one,6,8-di-b-Dglucopyranosyl-5,7-di-hydroxy-2-(3-hydroxy-4-me-thoxyphenyl)-	29.3	[M+H]+	625.1764	_	C_28_H_32_O_16_	_	✓	✓	✓	✓	✓	✓
104	*4H-1-Benzopyran-4-one, 2-phenyl-,dihyroxy trimethoxy deriv	38.79	[M+H]+	343.0662	191	C_14_H_14_O_10_	_	_	✓	_	_	✓	✓

Compounds labeled with asterisk (*) were newly generated during the sun-drying processing. Compounds labeled with star (*) means putative identification. “✓” means detected in this sample, and “_” means not detected in this sample.

The retention times (Rt), the ratio of deprotonated/protonated molecules ([M+H]^+^/[M-H]^−^), measured value, fragment ions and formula are listed in Table 2. Furthermore, some of the compounds in Table 2 were identified by standard comparisons, such as gallocatechin (GC), (-)-epigallocatechin (EGC), catechin (C), epicatechin (EC), epigallocatechin gallate (EGCG), gallocatechin gallate (GCG), epicatechin gallate (ECG), catechin gallate (CG), caffeine, theobromine, theophylline, gallic acid, rutin, quercetin, and synephrine.

As shown in Table 2, Compounds 1–72 were detected in Pu-erh raw tea (S1) and Ganpu tea (S3, S4, S5, S6, S7) except those labeled with asterisk, which were newly generated compounds during the sun-drying processing. For example, isoleucine, threonine, arginine, and glutamine were detected only from S3 (finished product), S6 (sun-drying for 9 days), and S7 (sun-drying for 15 days), and catechin-4a-epicatechin-3-O-gallate, catechin-4a-epicatechin 3'-o-gallate, myricetin−3- locust glycoside, quercetin−3-o- rhamnoside galactoside, and vitexin were detected only from S3 (finished product), S5 (sun-drying for 3days), S6 (sun-drying for 9 days), and S7 (sun-drying for 15 days), which means these compounds were produced at different times during the sun-drying processing. Meanwhile, compounds 73-104 were detected in mandarin peel (S2) and Ganpu tea (S3, S4, S5, S6, S7) except 4H-1-Benzopyran-4-one,2,3-dihydro-5,6,7,8-tetramethoxy-2-[4-(phenylmethoxy)phenyl]- and 4H-1-Benzopyran-4-one,2-phenyl-,dihyroxy trimethoxy deriv, which were detected only from S3 (finished product), S6 (sun-drying for 9 days), and S7 (sun-drying for 15 days).

As shown in Figure 1, the water-soluble chemical compounds of Pu-erh raw tea (S1), mandarin peel (S2), and Ganpu tea (S3) exhibited noticeable differences and intrinsic connections in all the chromatograms. The intensities of ionization varied frequently between the positive (Figure 1A) and the negative ion modes (Figure 1B), and the mechanisms also varied between MS (Figures 1A,B) and UV detectors (Figure 1C). Still, we found similar results, i.e., water-soluble chemical compounds of Ganpu tea (S3) were more abundant and diversified as compared with those of Pu-erh raw tea (S1) and mandarin peel (S2). A total of 104 compounds were detected in Ganpu tea (S3), while only 63 and 30 compounds were detected in Pu-erh raw tea (S1) and mandarin peel (S2), respectively. Water-soluble compounds of Ganpu tea (S3) were consisted of six amino acids, 16 catechins, three alkaloids, seven pigments, 19 organic acids, and 12 flavonoids, which were also detected in the Pu-erh raw tea (S1), and 30 compounds, which were also detected in the mandarin peel (S2). Furthermore, 11 compounds labeled with asterisk and listed in Table 2 were detected only in Ganpu tea (S3), which were newly generated during the sun-drying processing. The potential flavor compounds of Ganpu tea (S3) were shared with 59.6% compounds of Pu-erh raw tea (S1), and with 29.8% compounds of mandarin peel (S2). The remaining 10.6% potential flavor compounds in Ganpu tea were newly generated during the sun-drying processing.

### Analysis of Flavor Substances From Ganpu Tea

**Figure 2:**
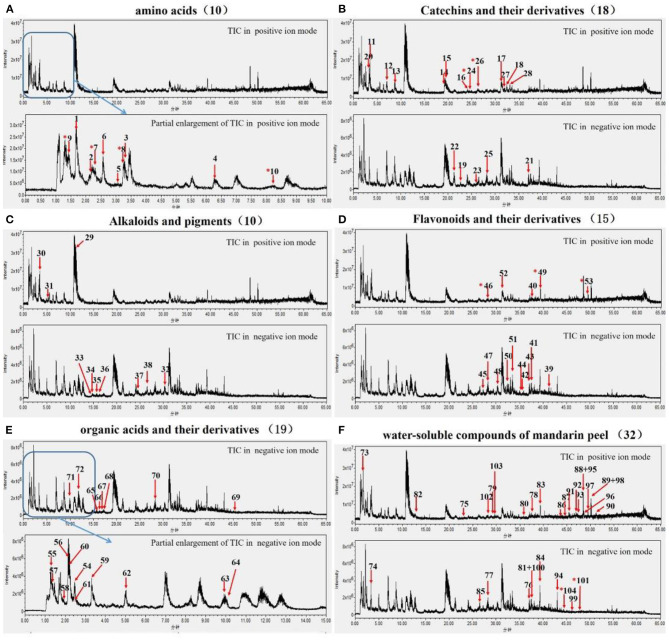
Flavor substances of Ganpu tea were identified by UPLC-Q-TOF system in different categories, including ten amino acids **(A)**, 18 catechins and their derivatives **(B)**, three alkaloids and seven pigments **(C)**, 15 flavonoids and their derivatives **(D)**, 19 organic acids and their derivatives **(E)**, and 32 water-soluble compounds of mandarin peel **(F)**, respectively.

A total of 104 potential flavor compounds were identified in Ganpu tea by UPLC-Q-TOF system. The characteristic peaks of these potential flavor compounds are classified in Figure 2, while other details are listed in Table 2. Our results showed that ten amino acids were identified in Ganpu tea. Theanine (1) is the most abundant compound, followed by pyroglutamic acid (6), threonine (8), and phenylalanine (3). Moreover, isoleucine (7), threonine (8), arginine (9), and glutamine (10), labeled with asterisks in Figure 2A, were newly generated amino acids during the full sun-drying processing, as they were identified exclusively in Ganpu tea, but not in Pu-erh raw tea or mandarin peel. In addition to the common tea catechins, such as GC (11), EGC (12), C (13), EC (14), EGCG (15), GCG (16), ECG (17), and CG (18), 10 other catechins and catechin metabolites were also identified in Ganpu tea. For example, epiafzelechin-3-O-gallate (21), gallocatechin-(4a-8)-catechin-3-O-gallate (22), and trigalloylglucose (24) all displayed a strong intensity of ionization. Notably, catechin-4a-epicatechin-3-O-gallate (25) and catechin-4a-epicatechin-3'-O-gallate (26) were newly generated during the sun-drying process, as they were discovered exclusively in Ganpu tea (Figure 2B). Caffeine (27) and theobromine (28) were the main alkaloids in Ganpu tea. The pigments were composed of theaflavin (29) and some anthocyanins (30–35) (Figure 2C). Flavonoids and their derivatives were identified in Ganpu tea and shown in Figure 2D. Rutin (36), kaempferol (37), myricetin-3-O-galactoside (47), quercetin (48), and their derivatives, which originated from Pu-erh raw tea were also identified in Ganpu tea. Three newly generated compounds, including myricetin−3- locust glycoside (46), quercetin−3-o- rhamnoside galactoside (49), and vitexin (53) were formed during the sun-drying processing. Meanwhile, flavonoids originating from mandarin peel are shown in Figure 2F. For example, naringenin (75), naringin (78), narirutin (81), hesperidin (84), poncirin (85), pentamethoxyflavanone (89), isosinensetin (91), sinensetin (92), limonin (94), nobiletin (95), and tangeretin (98) were the important chemical constituents for mandarin peel (24, 25). Nineteen organic acids and their derivatives (54–72) were identified and shown in Figure 2E.

As we known, catechins, pigments, amino acids, organic acids, alkaloids, and flavonoids were important for the flavor of tea drink and mandarin peel (24–26, 38). Therefore, we speculated that the unique flavor of Ganpu tea could co-attributed to these water-soluble compounds. Further researches are necessary to verify the 11 newly generated compounds whether also contribute to the flavor characteristic of Ganpu tea. The chemical compound identification of 104 metabolites of Ganpu tea was tentative in this paper. We need to positively identify major and effective compounds of Ganpu tea in our future research. Based on our previous study on the aroma of Ganpu tea (14), we predicted that potential flavor compounds would be changed considerably during the sun-drying processing due to *de novo* biosynthesis and/or enzymatic modification of the existing compounds, which were influenced by exogenous stimuli, including temperature, humidity, light, and microorganisms. To study the conversions of water-soluble compounds of Ganpu tea, we further analyzed the biochemical compositions in this study.

### The Variation Trend of Potential Flavor Compounds in Ganpu Tea During the Sun-Drying Processing

#### Analysis of Biochemical Components

Moisture, water extract, total free amino acids, tea polyphenols, theaflavins, thearubigins, and theabrownine have respective influences on the sensory flavor and health-promoting function of Ganpu tea. These primary biochemical components and their contents were assessed within four samples with different durations of sun-drying treatment, and listed in Table 3. Data were expressed from three independent chemical replicates. Sun-drying treatment for 15 days could significantly decrease the moisture contents from 24.16 to 5.04% of Ganpu tea, and the 15-day-period of sunlight was necessary to reach the final moisture content. It was clear that S4 without sun-drying treatment, contained the higher moisture contents, mainly due to the fresh mandarin peel of Ganpu tea. The sun-drying processing not only reduced the moisture content but also imposed significant effect on tea polyphenols and pigments. The concentration of tea polyphenols in S7 was significantly lower than those in other samples, due to the fact that catechins had undergone a series of oxidative, condensing and degradative chemical processes to form gallic acid, theaflavins, thearubigins, and theabrownine under the sun-drying conditions (11).

**Table 3:** Analysis of biochemical components of Ganpu tea samples with different durations of the full sun-drying processing.

**Biochemical component (%)**	**S4 (0 day)**	**S5 (3 day)**	**S6 (9 day)**	**S7 (15 day)**
Moisture	24.16 ± 1.38a	21.23 ± 1.04b	12.17 ± 0.76c	5.04 ± 0.98d
Water extract	30.04 ± 2.15d	31.72 ± 2.23cd	33.58 ± 2.02b	34.86 ± 1.93ab
Total free amino acids	2.37± 0.32a	2.03 ± 0.52b	2.33 ± 0.29a	2.43 ± 0.20a
Tea polyphenols	18.45 ± 1.02a	16.77 ± 0.15b	14.10 ± 1.13c	12.12 ± 1.42d
Theaflavins	0.24 ± 0.13a	0.15 ± 0.46b	0.13 ± 0.65bc	0.10 ± 0.02c
Thearubigins	1.36 ± 0.10c	1.49 ± 0.13b	1.50 ± 0.11b	1.85 ± 0.12a
Theabrownine	1.25 ± 0.17c	1.26 ± 0.03c	1.34 ± 0.20b	1.77 ± 0.21a

*Differences between samples were analyzed by Duncan's test. In each horizontal line, the mean ± SD values bearing different letters differ significantly (p < 0.05)*.

As shown in Table 3, at the end of sun-drying processing, the contents of thearubigins and theabrownine were clearly increased. On the contrary, the content of theaflavins was clearly decreased, suggesting that theaflavins are oxidized and accumulated into thearubigins and theabrownine. The content of total free amino acids was only slightly changed during the sun-drying processing since the contents of several free amino acids, including theanine and leucine, were decreased, while other amino acids, including isoleucine, threonine, arginine, and glutamine were newly generated. The total content of water extract was increased during the sun-drying processing, suggesting that new substances are formed from oxidation, degradation, and condensation of various compounds, which collectively contribute to the unique color, taste, and aroma of the Ganpu tea.

#### The Variation Trend of Potential Flavor Compounds

**Figure 3:**
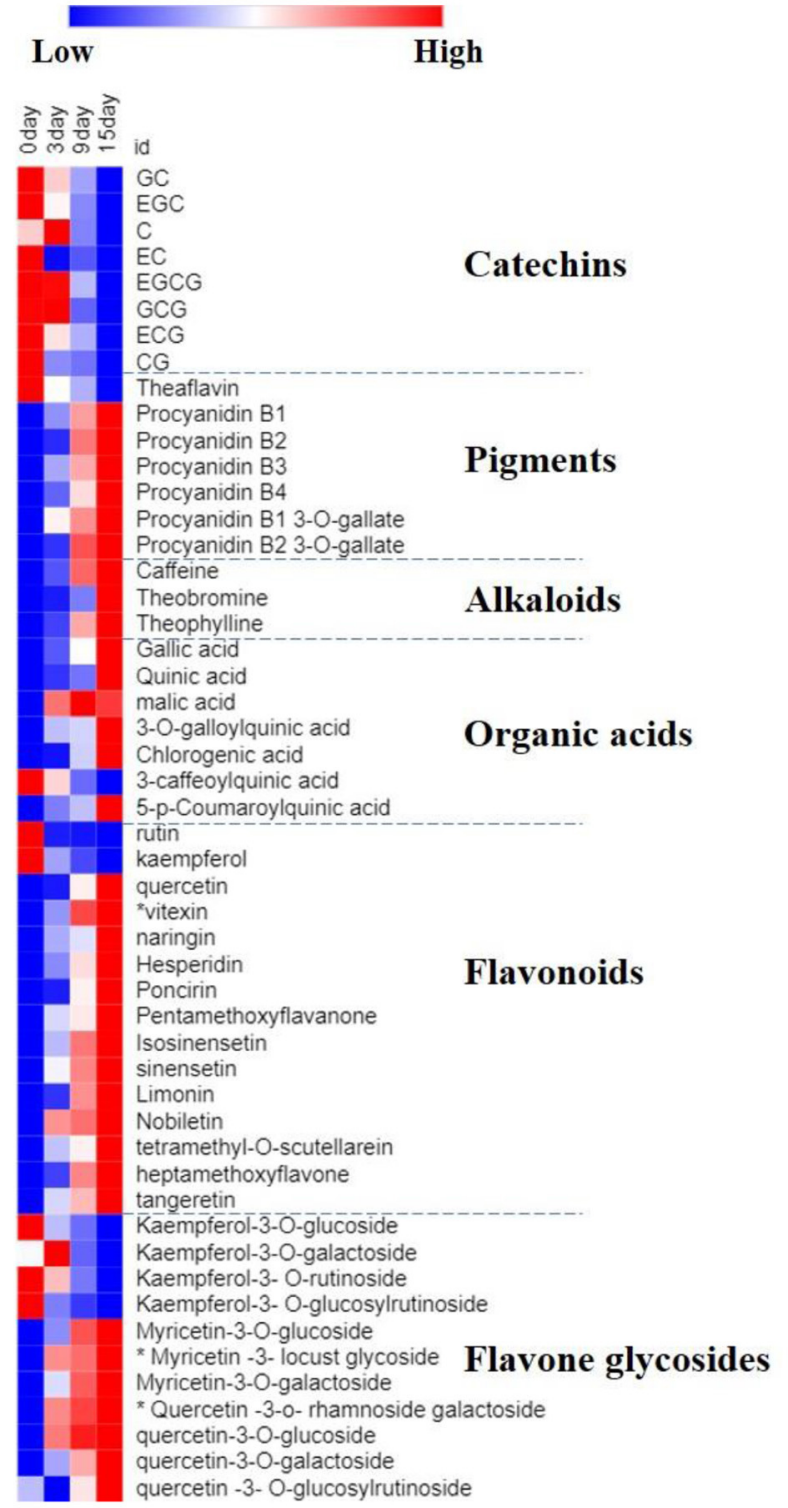
Heatmap analysis of potential flavor compounds from Ganpu tea.

A heatmap analysis was applied to visualize the dynamic changes of potential flavor compounds in Ganpu tea during the sun-drying processing (Figure 3). Each column represents a tea sample with different durations of sun-drying processing and each row represents a critical compound. A color-coded scale grading from blue to red corresponds to the content of these potential flavor compounds shifting from low to high. The sun-drying processing requires 15 days of high humidity and high temperature conditions, causing potential flavor compounds of Ganpu tea, including catechins, pigments, alkaloids, and flavonoids, to be transformed considerably.

Firstly, the contents of most catechins, particularly EGC, EGCG, GCG, and CG, were decreased by 20.2–27.2%. Meanwhile, with the exception of theaflavin, the pigments, including procyanidins and their derivatives, were clearly increased throughout the sun-drying processing. The significant reduction in catechin content from initial levels throughout the entire course of the sun-drying processing may be resulted from a series of chemical transformations of these compounds into gallic acid, pigments, and alkaloids, during the processes catalyzed by polyphenol oxidase and peroxidase enzymes (39).

Secondly, alkaloids were profoundly affected by the humid and hot sun-drying conditions. For example, the contents of caffeine, theobromine, and theophylline were increased considerably, with caffeine showing the most significant increase (28.7%). It has been proposed that extracellular enzymes produced by microorganisms, such as tannase, play a large role in catalyzing chemical reactions and promoting the transformation of the key chemical components (40).

Thirdly, organic acids showed different variation trends depending on their chemical compositions. For example, the content of 3-caffeoylquinic acid was decreased, while those of gallic acid, quinic acid, and chlorogenic acid were significantly increased with prolonging sun-drying time. Gallic acid is one of the most prominent phenolic acids in tea. The increase of its content during the sun-drying processing is likely due to the degradation of catechin gallates under high-temperature and high moisture conditions, which has been observed in a previous study (27).

Finally, a majority of the essential flavonoids identified in Ganpu tea were originated from the mandarin peel, and showed significant changes during the sun-drying processing. For instance, naringenin, naringin, narirutin, hesperidin, pentamethoxyflavanone, isosinensetin, sinensetin, limonin, nobiletin, and tangeretin were the crucial group of bioactive compounds in the mandarin peel, and they were all increased significantly during the sun-drying processing. Similarly, the contents of most common glycosidic group attached to the flavonoids in Ganpu tea were substantially increased as well. With the exception of kaempferol and its derivatives, whose contents were decreased, the contents of other flavone glycosides, including myricetin-3-O-galactoside and quercetin-3-O-galactoside, were increased significantly. Furthermore, the newly generated flavonoids, including myricetin-3-locust glycoside, quercetin-3-O-rhamnoside galactoside, and vitexin were detected starting from the third day of sun-drying processing. Biosynthesis and/or modification of constitutive flavonoids may be induced by exogenous stimuli, such as changes in sun light and temperature under the natural environments, as well as innate enzymatic activity of mandarin peel cells (28, 41). The persistent activities of enzymes involved in the flavonoid biosynthesis (chalcone flavanone isomerase, phenylalanine ammonia lyase, and cinnamate 4-hydroxylase), O-methylation enzymes (chalcone synthase, flavanone 7-O-glucosyltransferase, and flavanone 7-O-glucoside-2'-O-rhamnotransferase), methyltransferase, and chalcone-flavanone isomerase may be the important pathways for the accumulation and conversion of flavonoid compounds in Ganpu tea (29, 30, 39).

### DAS With a Trained Panel

**Figure 4:**
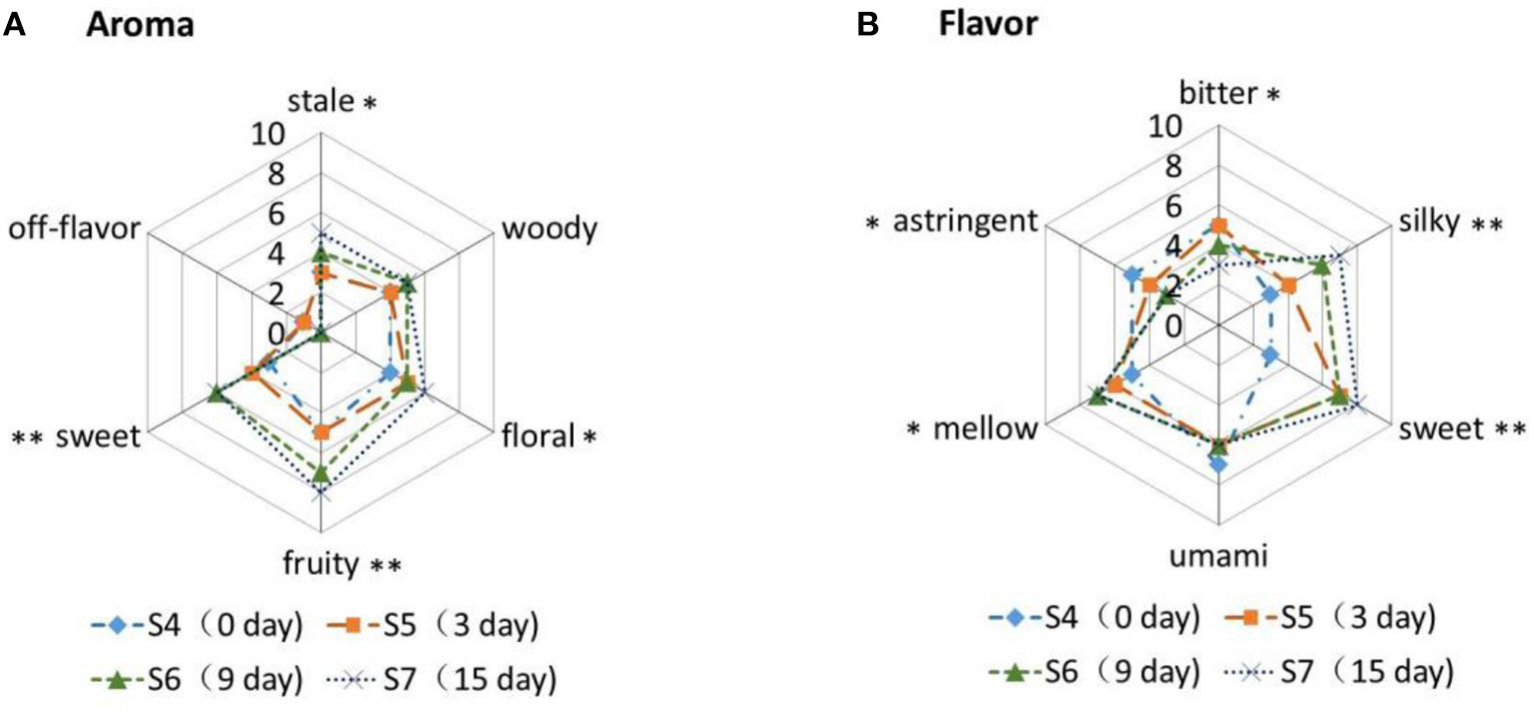
Aroma and flavor profiles of Ganpu tea samples with different durations of sun-drying processing. The statistical analyses were performed using ANOVA, followed by LSD test (*p < 0.05, **p < 0.01).

To date, the sensory characteristics of Gan*p*u tea have not been reported yet. In this study, we evaluated the influences of sun-drying processing on the sensory properties of Ganpu tea. The aroma and flavor attributes of Ganpu tea processed with different durations of sun-drying processing are presented in Figure 4. Six aroma attributes, including stale, woody, floral, fruity, sweet, and off-flavor, were evaluated in S4, S5, S6, and S7 (Figure 4A). The results showed that S7 demonstrated significantly higher intensities of sweet (*p* < 0.01) and fruity (*p* < 0.01) attributes. The 4-terpineol, thymol, and 4-isopropyl−3- methyl phenol contribute to the sweet descriptor while α-ionone, β-ionone, terpinene, p-acetylanisole, and isoamyl alcohol leaf ester contribute to the fruity descriptor (16). These compounds were accumulated in significantly higher quantities in Ganpu tea with longer duration of sun-drying treatments. S7 also displayed significantly higher intensities of stale (*p* < 0.05) and floral (*p* < 0.05) descriptors. Alcohols and ketones often contribute to the floral descriptor (31), and significantly higher quantities of linalool, 4-terpineol, pinocarvone, piperitone, carvone, and carvenone were detected in S7 (14). The off-flavor was detected in S4 and S5, which may be due to a flavor resembling fermentation of Pu-erh raw tea. However, the detection of off-flavor went away with prolonging the duration of sun-drying treatment.

The flavor attributes of bitter, silky, sweet, umami, mellow, and astringent were evaluated across the four treatment groups (Figure 4B). Although no significant differences (*p* > 0.05) in the umami descriptor were observed, S7 did demonstrate significantly higher intensities of silky (*p* < 0.01), sweet *(p* < 0.01), and mellow (*p* < 0.05) attributes. Soluble saccharides and certain amino acids, including theanine, phenylalanine, and tryptophan, contribute to sweetness. Moreover, flavonoids could provide a unique taste, changing from bitter to sweet following consumption (32). Additionally, the conversions of catechins, gallic acid, and caffeine as described in the previous section may result in a softer and mellower flavor. Interestingly, S7 displayed a significantly lower intensity (*p* < 0.05) of bitter and astringent attributes, which could be attributed to the significant decrease in catechin content over the course of the sun-drying processing (33).

In general, Ganpu tea has distinctive characteristics, including bright orange liquor, silky mellow taste, and fruity sweet aroma. The sun-drying approach significantly affected the intensities of the sensory attributes of Ganpu tea. Constituents of Ganpu tea are mostly polyphenols, flavonoids, caffeine, and free amino acids, and all of them were changed continuously throughout the sun-drying processing, most probably contributing to the unique taste developed during the Ganpu tea production.

### Effect of Ganpu Tea Consumption in the FST and TST

**Figure 5:**
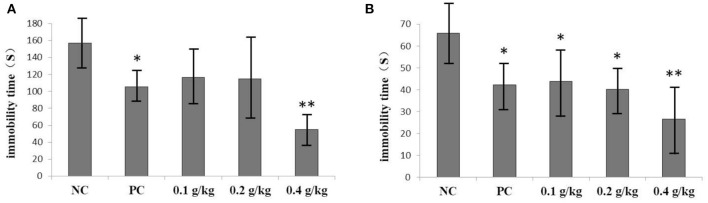
Effects of Ganpu tea on the immobility duration of mice in the behavioral tests. **(A)** FST, and **(B)** TST. Data are expressed as mean ± SD (*n* = 12 mice per group). NC, normal control group; PC, clomipramine hydrochloride tablets group (0.02 g/kg). The statistical analyses were performed using ANOVA, followed by LSD test, *p < 0.05, **p < 0.01 as compared with that of the NC group.

We hypothesized that tea polyphenols, flavonoids, pigments, theanine, caffeic acid, and vitexin detected in Ganpu tea in the present study would be collectively capable of conferring antidepressant-like effects through various mechanisms in animals, an effect that has been shown to take place following the regular consumption of most tea types (34). In order to test this hypothesis, we designed an *in vivo* experiment with mouse model, attempting to simulate the daily Ganpu tea consumption habits of humans. Both the FST and TST are the conventional experimental models of examining the depressive-like behaviors. The immobility times of these tests are shown in Figure 5. Results of the FST showed that no significant difference in immobility duration was seen between the NC group and the two oral dose groups (0.1 and 0.2 g/kg) of Ganpu tea extract (*p* > 0.05). The high oral dose group (0.4 g/kg) and PC group showed significantly reduced immobility duration as compared to that of the NC group (*p* < 0.01 and *p* < 0.05, respectively). TST results showed a dose-dependent reduction in immobility duration following intake of Ganpu tea extract. The effects of oral doses at 0.1 and 0.2 g/kg of Ganpu tea extract were similar to that of the PC group, which were significantly shorter than that of the NC group (*p* < 0.05). Meanwhile, high oral dose group (0.4 g/kg) recorded an immobility time of only 26.6 s, which was significantly shorter than that of the NC group (*p* < 0.01).

The significantly reduced immobility duration of the high oral dose group (0.4 g/kg) as compared to that of the NC group (*p* < 0.01) in the FST and TST suggests that the Ganpu tea extract produced antidepressant-like effects or otherwise helps to alleviate the depressive symptoms in mice. The anti-depressive activity of Ganpu tea was likely the collective or synergistic result of numerous beneficial compounds present in the Ganpu tea extract. Tea polyphenols may be related to the amelioration of monoaminergic responses, antioxidant defenses, and the hypothalamic-pituitary-adrenal axis (35, 36). Flavonoids, such as quercetin, rutin, kaempferitrin, and hesperidin in Ganpu tea can attenuate the damaged monoamine neurotransmission, including that of serotonin, noradrenaline, dopamine, and 5-hydroxyindoleacetic acid, and regulate the expression levels of genes encoding neurotransmitter receptors (37). Anthocyanins were shown to regulate the activation of noradrenergic, serotonergic, and dopaminergic system (37). Caffeic acid was suggested to be active within the dopaminergic system (37). Theanine can also similarly mediate the interactions within monoaminergic systems (42). It is noteworthy that vitexin is a newly generated compound in the sun-drying processing, which was shown by Can et al. (43) to increase the catecholamine content in the synaptic cleft and to interact with serotonergic 1A, noradrenergic a2, and dopaminergic D1, D2, and D3 receptors. Further studies are necessary to determine whether other compounds in Ganpu tea are also responsible for conferring the antidepressant-like effects, and what are the underlying molecular mechanisms.

## Conclusions

In the present study, we demonstrated that acquirement of the unique characteristics of high-quality Ganpu tea requires not only high-grade Pu-erh raw tea and mandarin peel, but also a full sun-drying processing for consecutive 15 days. The distinctive flavor of Ganpu tea was composed of a total of 104 water-soluble compounds, which were transformed considerably during sun-drying processing due to endogenous factors (biosynthesis or enzymatic modification of compounds within cells), as well as exogenous stimuli, including temperature, humidity, light, and microorganisms. The alterations in major chemical constituents contribute to the changes of aroma and taste of Ganpu tea. Meanwhile, tea polyphenols, flavonoids, pigments, theanine, caffeic acid, and vitexin may be also collectively responsible for the antidepressant-like effects of Ganpu tea consumption. These findings provide a basis or understanding the chemical characteristics and health-promoting effects of Ganpu tea, and also for elucidating its value as a new flavorful beverage with potential benefit for humans suffering from the depressive symptoms.

## Data Availability Statement

The raw data supporting the conclusions of this article will be made available by the authors, without undue reservation.

## Ethics Statement

All the procedures were conducted according to the National Institutes of Health Guide for the Care and Use of Laboratory Animals, and the animal handling and experimental procedures were approved by the Local Animal Use Committee (SCXK 2013-0020).

## Author Contributions

SX and XZ designed the concept of this study. SX, JH, HL, YZ, and DL drafted methodology and conducted formal analyses and investigation. HX and YH were project supervisors. Writing-Review and Editing were completed by HX, SX, JH, and YH. Funding Acquisition was by XZ. All authors contributed to the article and approved the submitted version.

## Conflict of Interest

HL, YZ, and DL were employed by Yunding Ganpu Tea Company. The remaining authors declare that the research was conducted in the absence of any conflict of interest.

## Abbreviations

GC: gallocatechin
EGC: (-)-epigallocatechin
C: catechin
EC: epicatechin
EGCG: epigallocatechin gallate
GCG: gallocatechin gallate
ECG: epicatechin gallate
CG: Catechin gallate
UPLC-Q-TOF: Ultra performance liquid chromatography coupled with Q-TOF mass spectrometry
UPLC-MS: Ultra-high performance liquid chromatography- mass spectrometry
Rt: retention time
DSA: descriptive sensory analysis
FST: forced swimming test
TST: tail suspension test.
